# Stuck Together: A Systematic Review of Hand Syndactyly

**DOI:** 10.7759/cureus.81943

**Published:** 2025-04-09

**Authors:** Shelleen Gowrie, Opeyemi Omosebi, Philip Veith, Neil Agarwal, Sameer Shah, Michael Montalbano, R. Shane Tubbs, Marios Loukas

**Affiliations:** 1 Anatomical Sciences, St. George's University, School of Medicine, St. George's, GRD; 2 Medicine, Danbury Hospital, Danbury, USA; 3 Internal Medicine, Northwell Health, Syosset Hospital, Syosset, USA; 4 Internal Medicine, University of Maryland Medical Center, Midtown Campus, Baltimore, USA; 5 Anatomy, St. George's University, School of Medicine, St. George's, GRD; 6 Neurosurgery and Structural and Cellular Biology, Tulane University, School of Medicine, New Orleans, USA; 7 Pathology, St. George's University, School of Medicine, St. George's, GRD

**Keywords:** genetic syndrome, musculoskeletal diseases, plastic surgery procedures, syndactyly, upper extremity

## Abstract

Hand syndactyly is a congenital malformation resulting from the failure of differentiation between two or more digits, resulting in a subsequent fusion. This systematic review aims to comprehensively analyze the genetic and embryological mechanisms underlying both syndromic and non-syndromic variants of hand syndactyly, as well as evaluate the effectiveness of surgical interventions utilized in the correction of this congenital anomaly to improve patient outcomes and reduce complications. The review follows Preferred Reporting Items for Systematic Reviews and Meta-Analyses (PRISMA) guidelines and systematically searched PubMed, Google Scholar, ScienceDirect, and Cochrane databases without time restrictions up to August 2024. Studies included in this review examined (1) genetic and embryological mechanisms of syndactyly; (2) classification systems; and (3) surgical treatment outcomes. Reference lists of identified articles were manually screened for additional studies. Exclusion criteria included (1) conference papers, letters to the editor, reviews, and video articles; (2) studies on congenital hand malformations other than syndactyly; (3) studies involving animal subjects; (4) studies with updated reviews from the same authors; (5) preliminary studies; and (6) studies with duplicate information. Out of 3015 initial records and 212 additional sources, 88 studies met the inclusion criteria, providing insights into the genetics, embryology, and surgical management of hand syndactyly. Risk of bias was assessed using the traffic light plot within the Robvis risk-of-bias framework. While many studies demonstrated methodological rigor based on the key domains assessed, limitations included recall bias in retrospective cohort studies, inconsistent stratification of surgical outcomes, and inadequate control for confounding factors. The findings emphasize the importance of early diagnosis and intervention, particularly surgical correction between 18 and 24 months, to improve functional and aesthetic outcomes. Various surgical techniques have been explored, each with distinct benefits depending on the type and extent of malformation. Patient-reported outcomes show higher satisfaction with upper extremity function and reduced pain interference, highlighting the value of personalized treatment approaches. Despite expanded classification systems and improved surgical techniques, the multifactorial etiology of hand syndactyly requires further investigation to refine diagnostic strategies and enhance treatment protocols, ultimately improving patient care.

## Introduction and background

Hand syndactyly is a congenital anomaly characterized by the fusion of two or more digits. Hand syndactyly is a complex and multifaceted condition, as it can occur in both syndromic and non-syndromic forms, has been linked to multiple genetic syndromes, and may require a staged surgical approach for cases involving extensive fusion [1]. Derived from the Greek words “sun,” meaning together, and “daktulos,” meaning digit, syndactyly results from the failure of differentiation between two or more adjacent digits during embryonic development. This condition presents a spectrum of severity, ranging from mild cases involving only interdigital webbing to more severe cases with fusion of bones, tendons, joints, muscles, and neuromuscular bundles [2]. It is the most common congenital hand malformation, with an incidence rate of one per 2000-3000 live births, with a higher incidence found among males [1-3].

The anatomical classification of syndactyly plays a key role in guiding treatment approaches. It can be categorized based on the structures involved. Simple syndactyly refers to the fusion of digits that involves only soft tissue. Complex syndactyly involves fusion that extends to adjacent bones. Complicated syndactyly is more severe than complex syndactyly and includes fusion of bones on surfaces other than the adjacent digit, sometimes with additional tissue beyond the cutaneous and bone structures.

Syndactyly can also be classified according to the degree of fusion. Complete syndactyly occurs when the fusion extends the entire length of the digit, including the nail folds. In contrast, incomplete syndactyly refers to cases where the fusion does not span the entire length of the digit [4].

Additionally, syndactyly can present in isolation, as part of a syndrome, or in conjunction with other abnormalities such as acrosyndactyly, cleft hand, or synpolydactyly. Acrosyndactyly occurs when the digits have separated normally during development, but the presence of an amniotic band surrounding them resulted in the fusion of the distal portion [5]. Cleft hand is when the central digit and metacarpal fail to form, resulting in a V-shaped cleft [6]. Synpolydactyly is the presence of additional digits in conjunction with excess webbing or fusion [7]. Syndactyly may present as a feature of different syndromes, including Apert, Poland’s, Pfeiffer, Jackson-Weiss, and Holt-Oram [8].

Surgical intervention is often recommended to improve hand function and aesthetics, with reconstruction focusing on the web space, lateral border of the digit, and nail fold [4]. While traditional techniques rely on skin grafts, newer methods aim to eliminate this necessity [9]. In bone fusion cases, proper skeletal alignment is crucial [10]. This systematic review aims to provide a comprehensive overview of hand syndactyly's current understanding and management.

## Review

Methods

Search Strategy

A systematic review was conducted to comprehensively examine the embryology, genetics, and surgical management of syndromic and non-syndromic hand syndactyly. The review adhered to the Preferred Reporting Items for Systematic Reviews and Meta-Analyses (PRISMA) guidelines. Literature searches were conducted through PubMed, Google Scholar, ScienceDirect, and the Cochrane databases without imposing temporal restrictions up to August 2024. The search strategy included the following Boolean search string for PubMed: ("hand syndactyly" AND (etiology OR pathogenesis OR classification OR embryology OR treatment OR "surgical outcomes"). Similar search strategies were applied to other databases. Duplicate studies were identified and removed using Microsoft Excel's conditional formatting function to maintain dataset integrity.

Eligibility Criteria

Studies included in this review examined (1) the genetic and embryological mechanisms underlying hand syndactyly; (2) classification systems; and (3) surgical treatment outcomes. Two reviewers (SG and OO) manually screened reference lists of identified articles, with conflicts resolved through discussion with additional reviewers (MM and ML), to identify studies meeting the inclusion criteria.

Studies were limited to those published in English as part of the initial search strategy. The exclusion criteria were as follows: (1) conference papers, letters to the editor, reviews, and video articles; (2) studies addressing congenital hand malformations other than syndactyly; (3) studies involving animal subjects; (4) studies with updated reviews from the same authors; (5) preliminary studies; (6) non-English studies; and (7) studies with duplicate information.

Study Identification

**Figure 1:**
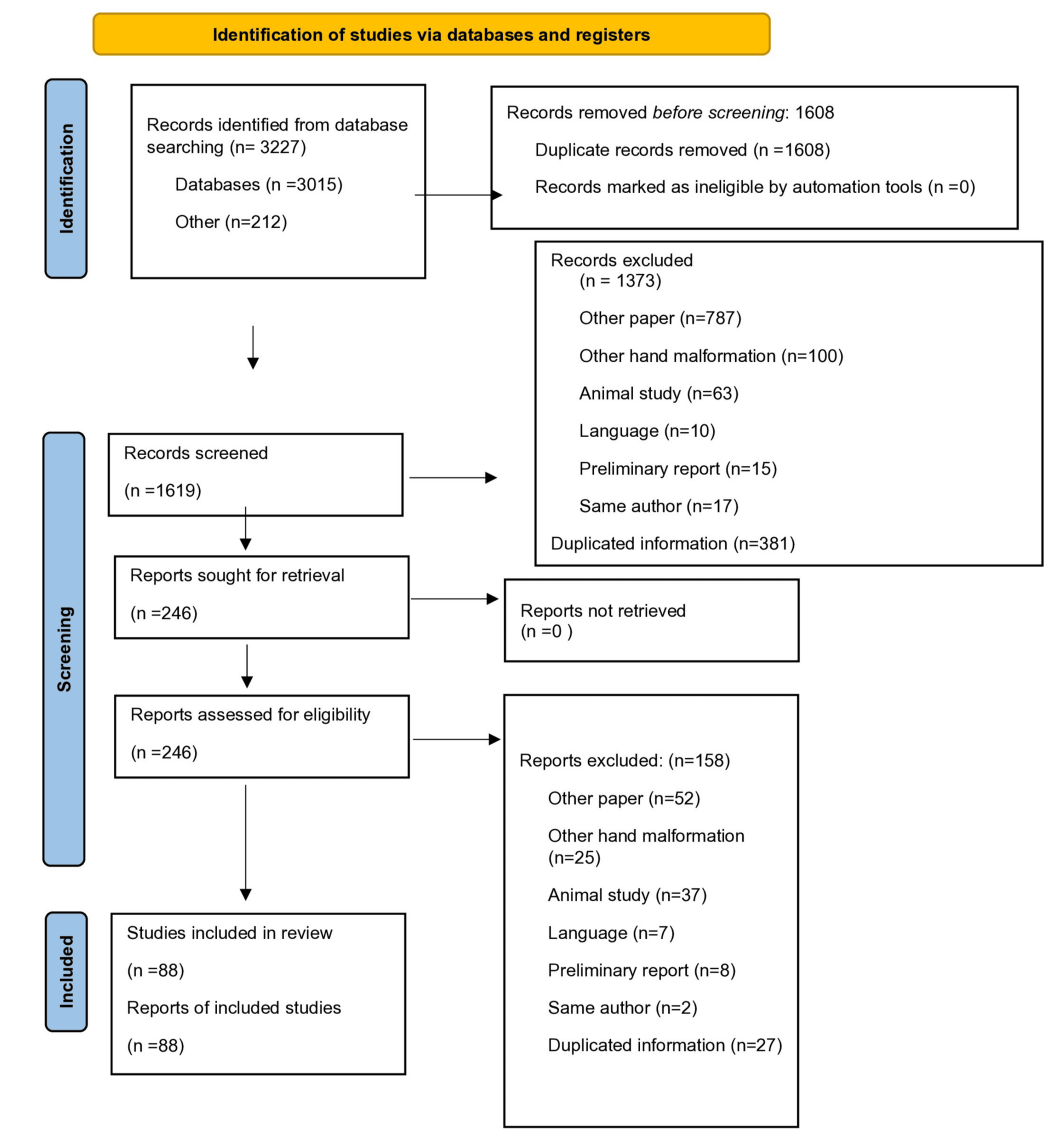
PRISMA flow diagram depicting the systematic review process for hand syndactyly studies PRISMA: Preferred Reporting Items for Systematic Reviews and Meta-Analyses

The search initially yielded 3015 studies, supplemented by 212 additional records identified through reference screening. Following deduplication, 420 articles were removed. Title and abstract screening led to the exclusion of 1373 articles due to non-relevance, resulting in 246 full-text articles being assessed for eligibility. Further exclusions included 52 articles due to non-relevant publication types, 25 articles focusing on other hand malformations, 37 studies involving animal subjects, seven non-English studies, eight preliminary reports, two studies that were updated articles by the same authors, and 27 studies that had duplicated information. Ultimately, 88 studies met the inclusion criteria and were incorporated into the final analysis. A PRISMA flow diagram illustrates the study selection process (Figure 1).

Risk of Bias Assessment

The risk of bias in the included studies was assessed using Robvis, a validated tool for visualizing bias in non-randomized studies [11]. As utilized in the Cochrane risk-of-bias framework, the traffic light plot approach facilitated a systematic evaluation of methodological quality. The traffic light plot presents a color-coded summary of bias judgments across multiple domains, highlighting areas of methodological strength and concern. Key domains assessed included sample size adequacy, selective outcome reporting, and control of confounding variables.

Results

Study Quality Assessment

**Figure 2:**
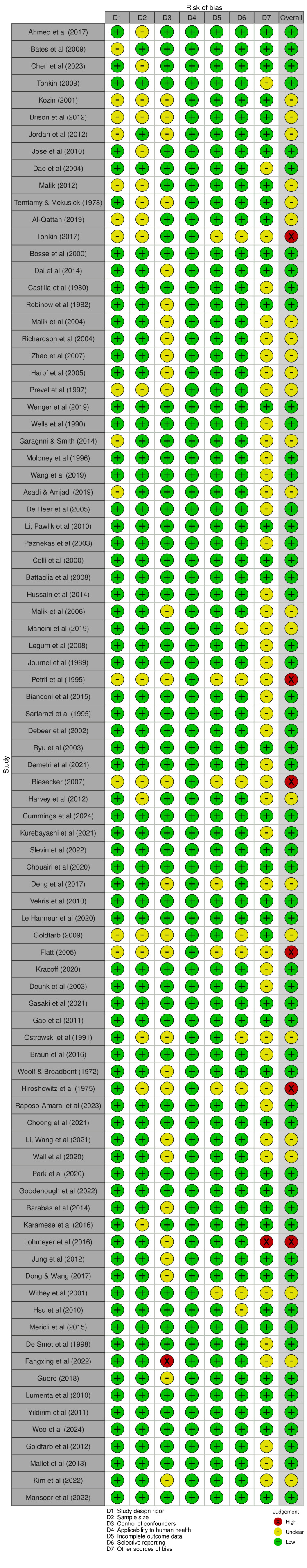
Traffic light plot of included studies A traffic light plot illustrating the risk of bias assessment for all 88 studies included in this systematic review on hand syndactyly [10].

**Figure 3:**
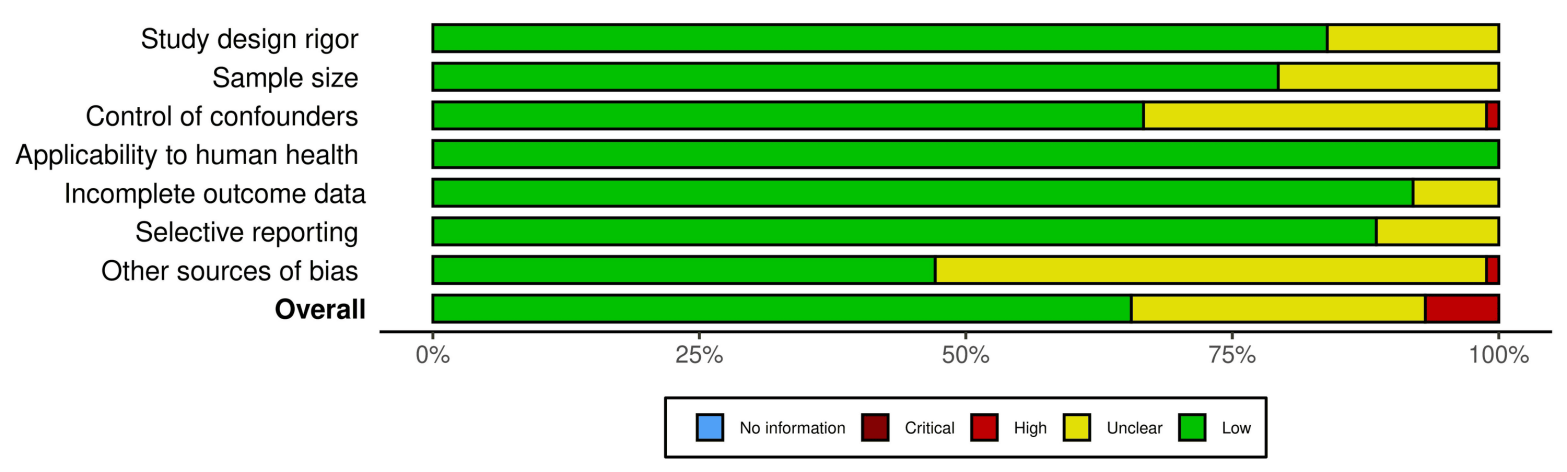
Summary of risk of bias across studies A summary figure consolidating the traffic light plot results, providing an overall assessment of the methodological quality of included studies [10].

The risk of bias assessment, summarized in Figure 2 and Figure 3, revealed considerable variation in methodological quality among the included studies. The traffic light plot highlighted differing bias levels across key domains, including study design rigor, sample size adequacy, and potential conflicts of interest. While most studies exhibited transparency and methodological soundness, certain limitations were evident.

Several studies, particularly genetic association analyses and surgical outcome evaluations, featured robust sample sizes, enhancing the reliability of their findings. However, methodological constraints were noted in others. Despite providing valuable long-term outcome data, retrospective cohort studies were inherently susceptible to recall bias. Additionally, some cohort studies failed to adequately control for confounding factors, such as distinguishing between complex and simple syndactyly cases. Furthermore, in studies employing multiple surgical techniques, results were often reported collectively rather than stratified by approach, limiting direct comparisons and the ability to assess technique-specific outcomes.

Non-syndromic Syndactyly

**Figure 4:**
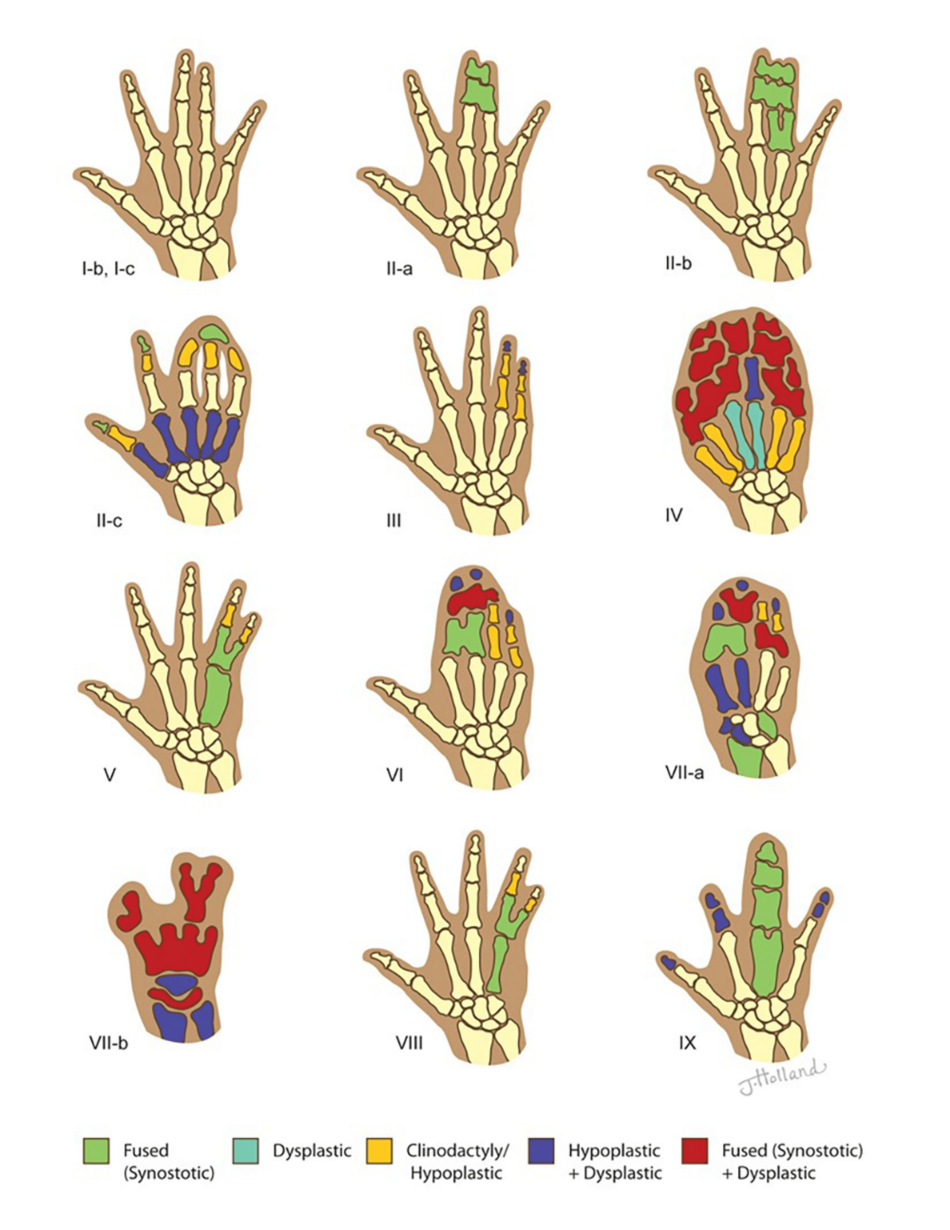
Illustration of non-syndromic hand syndactyly classifications Image Credit: This original illustration was created by Jessica Holland MSMI CMI for the authors. This image was created using traditional drawing tools and then later rendered in Adobe Photoshop and labeled in Adobe Illustrator (© CC-BY-ND 2025 Jessica Holland MSMI CMI, St. George's University).

A total of nine studies reported on non-syndromic syndactyly, identifying nine distinct subtypes. Type I was the most frequently reported, characterized by fusion of the third and fourth fingers or second and third toes. Type II was often associated with polydactyly, while Types III-V displayed varying degrees of digit and metacarpal fusion. The rarer forms (Types VI-VIII) demonstrated unique features, including unilateral syndactyly and uncertain inheritance patterns. Type VII (Cenani-Lenz syndrome) and Type IX (mesoaxial synostosis syndactyly) were notable for complete bony fusion and skeletal abnormalities. A detailed breakdown of the types, including prevalence and genetic associations, is provided in Table 1 (see Figure 4 for representative cases).

**Table 1:** Genetic loci and features of non-syndromic syndactyly types

Reference	Syndactyly	Locus	Gene	Inheritance pattern	Description/location of deformity
Syndactyly Type I: zygodactyly					
Jordan et al. (2012) [8]	I-a: Weidenreich type	3p21.31	-	Autosomal dominant	2nd and 3rd toes
Bosse et al. (2000) [12]	I-b: Lueken type	2q34-q36	-	Autosomal dominant	3rd and 4th fingers as well as 2nd and 3rd toes
Dai et al. (2014) [13]	I-c: Montagu type	﻿2q31-q32	﻿HOXD13	Autosomal dominant	3rd and 4th fingers
Malik (2012) [14]	I-d: Castilla type	-	-	Autosomal dominant	4th and 5th toes
Syndactyly Type II: synpolydactyly					
Malik (2012) [14]	II-a: Vordingborg type	2q31	HOXD13	Autosomal dominant	3rd and 4th fingers and 4th and 5th toes
Jordan et al. (2012) [8]	II-b: Debeer type	22q13.3	FBLN1	Autosomal dominant	3rd and 4th fingers and 4th and 5th toes
Malik (2012) [14]	II-c: Malik type	14q11.2-q13	-	Autosomal dominant	3rd and 4th fingers and 5th toe
Malik (2012) [14]; Richardson et al. (2004) [15]	Syndactyly Type III: Johnston-Kirby type	6q21-q23	GJA1	Autosomal dominant	3rd and 4th with smaller 5th finger
Syndactyly Type IV					
Jordan et al. (2012) [8]	IV-a: Haas type	7q36	ZRS (LMBR1)	Autosomal dominant	Cup-shaped hand
Malik (2012) [14]	IV-b: Andersen-Hansen type	-	-	-	Cup-shaped with foot involvement
Robinow et al. (1982) [16]; Zhao et al (2007) [17]	Syndactyly Type V: Dowd type	2q31	HOXD13	Autosomal dominant	Metacarpal fusion of 4th and 5th fingers and mesoaxial webbing of the feet
Jordan et al. (2012) [8]	Syndactyly Type VI: Mitten type	-	-	Autosomal dominant	Between 2nd and 5th fingers and the 2nd and 3rd toes
Syndactyly Type VII					
Malik (2012) [14]; Harpf et al. (2005) [18]	VII-a: Cenani-Lenz type	11p12-p11.2	LRP4	Autosomal recessive	Spoonhead shaped
Malik (2012) [14]	VII-b: Oligodactyly type	15q13.3	GREM1-FMN1	Autosomal dominant	Complete synostosis of the hand with metacarpal fusion and metatarsal fusion
Syndactyly Type VIII					
Jordan et al. (2012) [8]	VIII-a: Orel-Holmes type	Xq26	MF4	Autosomal dominant	Metacarpal fusion of 4th and 5th fingers
Jordan et al. (2012) [8]	VIII-b: Lerch type	﻿Xq21.1	﻿FGF16	Autosomal dominant	Metacarpal fusion of 4th and 5th fingers
Jordan et al. (2012) [8]; Malik (2012) [14]; Malik et al. (2004) [19]	Syndactyly Type IX: MSSD; Malik-Percin type	17p13.3	-	Autosomal recessive	Synostosis between the 3rd and 4th fingers and preaxial webbing of the feet

Syndromic Syndactyly

Syndromic syndactyly was described in 15 studies, with acrocephalosyndactyly syndromes (Apert, Carpenter, Pfeiffer, Jackson-Weiss, and Saethre-Chotzon) being the most frequently reported. FGFR mutations were the predominant genetic cause, except in Carpenter syndrome (RAB23) and Saethre-Chotzon syndrome (TWIST1). Other syndromes, such as Goltz syndrome, ectrodactyly-ectodermal dysplasia-clefting (EEC), and oculodentodigital dysplasia, were less frequently documented but exhibited distinct clinical patterns. A summary of syndromic syndactyly subtypes and their associated genetic and phenotypic characteristics is presented in Table 2.

**Table 2:** Genetic loci and features of syndromic syndactyly types

Reference	Syndromes	Loci	Gene	Inheritance pattern	Description/location of deformity
Acrocephalosyndactyly					
Prevel et al. (1997) [20]; Wenger et al. (2019) [21]; Moloney et al. (1996) [22]	Apert syndrome	﻿10q26.13	FGFR2	Autosomal dominant	Type1: 2nd-4th fingers, Type 2: 2nd-5th fingers, Type 3: all fingers
Garagnani and Smith (2014) [23]	﻿Carpenter syndrome	﻿6p11.2	﻿RAB23	Autosomal recessive	Syndactyly, polydactyly, and brachydactyly of the hands and feet
Wang et al. (2016) [24]	﻿Jackson-Weiss syndrome	﻿8p11.23	﻿FGFR1	Autosomal dominant	2nd and 3rd toes
		10q26.13	FGFR2	Autosomal dominant	2nd and 3rd toes
Asadi and Amjadi (2019) [25]	﻿Pfeiffer syndrome	﻿8p11.23	﻿FGFR1	Autosomal dominant	﻿Partial fingers and toes syndactyly with hands and feet brachymesophalangia
		﻿10q26.13	FGFR2	Autosomal dominant	Partial fingers and toes syndactyly with hands and feet brachymesophalangia
de Heer et al. (2005) [26]	Saethre-Chotzon syndrome	﻿7p21.1	﻿TWIST1	Autosomal dominant	2nd and 3rd fingers
		﻿10q26.13	﻿FGFR2	Autosomal dominant	3rd and 4th toes
Garagnani and Smith (2014) [23]	Bardet-Biedl syndrome	21 loci	21 genes	Autosomal recessive	Variable
Garagnani and Smith (2014) [23]; Li et al. (2010) [27]	﻿Cenani-Lenz syndactyly syndrome	﻿11p12-p11.2	﻿LRP4	Autosomal recessive	Bilateral complex syndactyly of the hands, “spoon-hand,” and feet.
Paznekas et al. (2003) [28]	﻿Ectrodactyly-ectodermal dysplasia-cleft lip/palate syndrome 1	﻿7q11.2-q21.3	﻿ -	Autosomal dominant	Syndactyly with ectrodactyly of hands or feet
Celli et al. (2000) [29]	Feingold syndrome 1	2p24.3	MYCN	Autosomal dominant	2nd and 3rd, 4th and 5th toes
Garagnani and Smith (2014) [23]	Fraser syndrome 1	4q21.21	FRAS1	Autosomal recessive	Cutaneous syndactyly
	Fraser syndrome 2	13q13.3	FREM2	Autosomal recessive	Cutaneous syndactyly
	Fraser syndrome 3	﻿12q14.3	GRIPI	Autosomal recessive	Cutaneous, partial, and bilateral
Battaglia et al. (2008) [30]; Hussain et al. (2014) [31]	﻿Filippi syndrome	2q14.1	CKAP2L	Autosomal recessive	3rd and 4th fingers, 2nd-4th toes
Paznekas et al. (2003) [28]	﻿Goltz syndrome	﻿Xp11.23	﻿PORCN	Sporadic/X-linked	Syndactyly of hands and feet with split hand and hypoplastic phalanges/metacarpals. Combination resembles “lobster hand.”
Battaglia et al. (2008) [30]; Hussain et al. (2014) [31]	﻿Greig cephalopolysyndactyly syndrome	7p13	GLI3	Autosomal dominant	3rd and 4th fingers, 1st-3rd toes
Malik et al. (2006) [32]	﻿Holt-Oram syndrome	12q24.21	﻿TBX5	Autosomal dominant	Syndactyly of the hand with characteristic thumb anomaly
Garagnani and Smith (2014) [23]	McKusick-Kaufman syndrome	20p12.2	MKKS	Autosomal recessive	Variable presentation
Mancini et al. (2018) [33]	﻿Microphthalmia with limb anomalies	14q24.2	﻿SMOC1	Autosomal recessive	4th and 5th metacarpophalangeal synostosis
Legum et al. (1981) [34]; Journel et al. (1889) [35]	﻿Moebius syndrome	13q12.2-q13	-	Sporadic/autosomal dominant	Syndactyly with hand deformities such as brachydactyly, and low-set thumb
	﻿Oculodentodigital dysplasia	﻿6q22.31	GJA1	Autosomal dominant	4th and 5th fingers, 3rd and 4th toes
Petrii et al. (1995) [36]	﻿Pallister-Hall syndrome	﻿7p14.1	﻿GLI3	Autosomal dominant	Unilateral, fingers only
Garagnani and Smith (2014) [23]	﻿Poland syndrome	-	-	Autosomal dominant	Syndactyly with brachydactyly, and oligodactyly (mostly right-sided)
Petrii et al. (1995) [36]	﻿Rubinstein-Taybi syndrome	16p13.3	﻿CREBBP	Autosomal dominant	Fingers only
Bianconi et al. (2015) [37]	﻿Smith-Lemli-Opitz syndrome	﻿11q13.4	DHCR7	Autosomal recessive	2nd and 3rd toes
Mancini et al. (2018) [33]	Short-rib thoracic dysplasia 7	2p24.1	WDR35	Autosomal recessive	variable presentation
Sarfarazi et al. (1995) [38]; Debeer et al. (2002) [39]; Malik et al. (2006) [32]	Synpolydactyly 1	2p31.1	HOXD13	Autosomal dominant	3rd and 4th fingers, 4th and 5th toes
	Synpolydactyly 2	22q13.31	FBLN1	Autosomal dominant	3rd and 4th fingers, 2nd-4th toes
	﻿ Synpolydactyly 3	14q11.2-q12	-	Autosomal dominant	Simple syndactyly
Celli et al. (2000) [29]	﻿Timothy syndrome	﻿12p13.33	CACNA1C	Autosomal dominant	Cutaneous hand and foot syndactyly
Mancini et al. (2018) [33]	Triphalangeal thumb-polysyndactyly syndrome	7q36.3	LMBR1	Autosomal dominant	Variable presentation

Surgical Techniques in Syndactyly Repair

A total of 14 studies reported on various flap techniques used in syndactyly repair, highlighting differences in surgical approach, complication rates, and functional outcomes. Table 3 presents a comparative analysis of various surgical techniques utilized to reconstruct syndactyly, focusing on their outcomes and associated complications. The selected studies highlight a diverse range of approaches, including flap-based techniques with and without the use of skin grafts, as well as modifications aimed at improving web space reconstruction and minimizing postoperative complications such as web creep, contractures, and scarring. Key considerations in evaluating these techniques include operative time, need for secondary interventions, aesthetic outcomes, functional recovery, and long-term stability of the reconstructed web space. This comparative assessment provides insights into the evolution of surgical strategies for syndactyly repair and underscores the importance of technique selection based on case complexity and patient-specific factors.

**Table 3:** Comparative analysis of surgical techniques and the outcomes for syndactyly reconstruction

Article	Technique	Number of webs	Outcomes
Whitey et al. (2001) [40]	Dorsal rectangular flap with interdigital zigzag	12	A combination of dorsal rectangular flaps with interdigitating zigzags was used to create 7–8 flaps that were not defatted. Postoperatively, the wounds healed by secondary intention, eliminating the need for grafts. No ischemia, necrosis, or digit contracture was observed during the postoperative period in any of the 12 cases. At follow-up, the "open" group, whose wounds were left to heal by secondary intention, demonstrated significantly less scarring and contracture compared to the control group (dorsal rectangular flaps with full-thickness grafts).
Hsu et al. (2009) [41]	Modified dorsal V-Y flap	28	Modification of the dorsal V-Y flap involves reshaping the flap on the dorsum of the hand to be more hexagonal. This allows the tip of the flap to approximate more easily at the newly created webspace without torsion, thereby reducing the risk of ischemia or necrosis. It also avoids the need for grafts by utilizing a local flap. Operation time was significantly shorter compared to traditional dorsal flap procedures with full-thickness graft repairs. However, this technique is best suited for simple syndactyly cases, as complex cases may still require grafting due to insufficient skin to cover the lateral borders.
Vekris et al. (2010) [42]	Dorsal rectangular flap with two volar triangular flaps	114	Ninety-seven percent of patients had good to excellent results, with a natural appearance and minimal web migration (<6 mm). Only three cases (2.6%) required revision due to web creep and palmar junctional scarring. The nail folds had a satisfactory appearance.
Vekris et al. (2010) [42]	Two triangular flaps	11	Seven cases (63%) developed web creep and palmar junctional scarring, requiring revision using a dorsal rectangular flap and two triangular volar flaps.
Jose et al. (2010) [9]	Combination flap technique with zigzag technique interdigital	221	Postoperatively, a 6% complication rate was observed. Web creep occurred in 5% of cases (only in complex syndactyly). Three percent of the cohort required reoperation. At the mean two-year follow-up, seven cases developed contractures, and five required revisions. No skin grafts were needed in 11 cases of incomplete simple syndactyly.
Lumenta et al. (2010) [43]	Dorsal triangular flap with zigzag technique	26	There were two cases of minor recurrence, 13 cases with normal soft web comparable to the contralateral unaffected hand, and 11 cases with web thickening. Regarding skin matching and pigmentation, six cases showed normal pigmentation, while 16 exhibited hypopigmented scarring. Eight patients demonstrated normal scar pliability post-operatively, and 12 had supple pliability. Notably, in this study, 17 out of 24 cases involving full-thickness skin grafting exhibited hair growth in the grafted areas postoperatively. There was no statistically significant difference between the operated hand and the contralateral unaffected hand in terms of range of motion, deformity, or contractures. No patient reported experiencing excessive sweating, itching, or pain postoperatively.
Yildirim et al. (2011) [44]	Dorsal V-Y with volar triangular flap and digital zigzag technique	15	The operative time for this procedure was approximately 60 minutes. In the immediate post-operative period, no patients experienced acute complications such as infection, graft failure, or hematoma. Follow-up ranged from six to 24 months, with none of the patients developing web creep. All patients reported good functional and aesthetic outcomes, including inconspicuous scars with normal pigmentation. No revisional surgeries were required, and all patients reported good-to-excellent satisfaction.
Gao et al. (2011) [45]	Dorsal pentagonal local flap	17	There were no intraoperative or acute complications in the immediate postoperative period. Complete healing occurred within two weeks for all cases, with none of the patients developing web creep, flap loss, hypertrophic scarring, or contractures at the mean 26-month follow-up. Most patients had acceptable scars with normal pigmentation. None required secondary operations, and parents reported high satisfaction.
Mericli et al. (2015) [46]	M-to-V flap	89	In this study, 14% of cases experienced complications, with 9% developing web creeps postoperatively. Seven percent of the cohort required surgical revision, and a higher complication rate was observed with longer follow-up durations (>2 years: 28% complication rate). However, all patients retained full hand function at the final follow-up.
Karamese et al. (2015) [47]	V-Y and rectangular flap	16	A combination of dorsal rectangular and volar V-Y flaps was used for the creation of a natural web space. The postoperative period was uneventful in all 16 patients. There were no postoperative ischemia, hematomas, infections, flap necrosis, graft failures, or reoperations. Fourteen patients had perfect postoperative evaluations, with good range of motion, no scarring, and no hyperpigmentation observed at the two-year follow-up. However, two patients had hyperpigmented areas noted in their skin grafts in the interdigital space. They also had minimal elastic scarring with a scar height of 1 mm.
Braun et al. (2016) [48]	Trilobed flap	Not specified	There is no contracture at follow-up. Tissue is taken from adjacent digits to prevent proximal extension of the dorsal incision.
Braun et al. (2016) [48]	Three-square-flap	Not specified	Achieves circumferential coverage without the need for a skin graft. Suitable for cases without extensive soft tissue deficiency.
Dong and Wang (2017) [49]	Dorsal double wing flap	35	The mean operative time was approximately 45 minutes. No patients developed complications in the postoperative period, including hematoma, infection, or flap necrosis. With this technique, there was no need for skin grafts. At follow-up, there was a 3% incidence of web creep (1 case, revised without grafts). However, there was no hypertrophic scarring, keloid formation, or contractures. Overall, patients had good aesthetic outcomes with inconspicuous scars.
Guero (2019) [50]	Modified trident flap	127	No flap necrosis. Three cases of Grade 1 web creep and two cases of Grade 2 web creep (requiring revision surgery). One minor and one intermediate contracture required revision. Postoperative abduction angles were >30° in all cases. High satisfaction (mean score of 1.1/10 on the VAS). Long-term follow-up (15+ years) showed no web creep and nearly invisible incisions, even in individuals with darker skin tones.
Raposo-Amaral et al. (2023) [51]	Quadrangular flap	Approximately 313 webs (93 patients with approximately 3.37 operations per patient)	Used for Apert syndrome Type III, it involves quadrangular flaps. There was no digit loss perioperatively. No major complications were reported, except for partial/total skin graft loss, which Raposo-Amaral et al. (2023) did not consider a complication. Eighty-seven percent of Type III cases achieved a five-digit hand, while 13% achieved a four-digit hand. Independent digit movement was achieved in all cases. Fourteen revisional surgeries were performed to deepen the commissure.

Advantages and Disadvantages of Surgical Techniques for Syndactyly Repair

Syndactyly repair remains a complex challenge in reconstructive surgery, with various surgical techniques employed to achieve optimal functional and aesthetic outcomes. A key goal of these procedures is to restore independent digit movement and prevent complications such as web creep, scarring, and contracture. Several techniques have been developed and refined over the years, each with distinct advantages and drawbacks depending on the clinical presentation and severity of the syndactyly. The following table summarizes the key findings from the reviewed studies, comparing the advantages and disadvantages of these techniques, with particular emphasis on the incidence of web creep, reoperation rates, and long-term results (Table 4).

**Table 4:** Advantages and disadvantages of surgical techniques for syndactyly repair FTSG: full-thickness skin grafting, DIP: distal interphalangeal, PIP: proximal interphalangeal

Article	Technique	Procedure design	Advantages	Disadvantages
Jose et al. (2010) [9]	Palmar flap, V-flaps, and reciprocal V-flaps	Uses a palmar flap with a "gothic arch" for web space reconstruction. V-flaps and full-thickness grafts cover lateral defects. Reciprocal V-flaps create nail folds, concealing scars in adduction	Aesthetic and functional results are excellent. Scars and skin grafts are concealed in adduction. Unique palmar flap design improves the span of the hand and provides functional benefits, especially in small hands.	Requires full-thickness skin grafts in complex syndactyly. Contractures and web space creep (5%) in complex cases. Some complications like partial graft loss, nerve injury, and contractures require revisions (3% re-operation rate).
Al-Qattan (2001) [52]	Dorsal rectangular flap with interposing triangular digital flaps	Dorsal rectangular flap with triangular digital flaps. Split-thickness grafts from buttock/thigh. Neurovascular dissection under magnification. Metacarpal ligaments divided for deeper webs.	Simple and reduced surgery time. Better graft take compared to full-thickness grafts. No major complications (e.g., no infection or necrosis).	The cosmetic appearance of split-thickness grafts is inferior to full-thickness grafts. Graft contraction can contribute to web creep (25% of cases). Hyperpigmentation of grafts.
Vekris et al. (2010) [42]	Dorsal rectangular and two volar triangular flaps	Dorsal and volar triangular flaps reconstruct web spaces, including polydactyly/clinodactyly corrections. Full-thickness skin grafts used.	High rate of good to excellent results (97%). Minimal migration (less than 6mm) of the web and rare palmar junctional scars. Natural appearance of the web with satisfactory nail folds. Effective correction of malformations and functional impairments in syndactyly, polydactyly, and clinodactyly. Secondary surgeries allowed refinement of functional and cosmetic outcomes. Effective in cases with complex syndactyly.	Some cases required multiple surgeries (up to four), increasing the overall treatment burden. Partial epidermolysis of grafts occurred in four cases, requiring conservative management. Complications included temporary blood supply disturbances in the ring digit (managed with aspirin) and hypoplasia of neurovascular bundles in complex syndactylies. Early technique with two triangular flaps abandoned due to unsatisfactory results.
Gao et al. (2011) [45]	Dorsal pentagonal local flap	Dorsal pentagonal flap advanced to reconstruct the web, eliminating grafts in most cases. Zigzag incisions used for digit resurfacing.	All 17 web releases in 10 patients were completed without intraoperative complications. The dorsal flaps remained viable, with no flap loss, wound infection, or breakdown. Complete healing occurred within two weeks, and the reconstructed webs exhibited normal skin quality, color, and contour, with no flexion or extension contractures. The use of local dorsal skin eliminated the need for skin grafts in most cases, and scarring was minimal and aesthetically acceptable. The technique was suitable for reconstructing multiple non-adjacent webs in a single procedure, and no cases of web creep or need for revision surgery were observed during the mean 26-month follow-up.	Limitations of this technique included a relatively short follow-up duration, which precluded definitive conclusions on long-term web creep. Additionally, most cases involved simple syndactyly, with only one patient presenting complex syndactyly, thereby limiting the generalizability of outcomes to more complex presentations.
Braun et al. (2016) [48]	Reverse W-M flap	Dorsal bisected triangular flaps interdigitated with similar palmar flaps to create the web space.	No web creep observed.	Skin grafting led to contracture in <1% of patients, requiring release by Z-plasty.
Braun et al. (2016) [48]	M-V flap	Dorsal V-shaped flap is transposed into the cleft of a palmar M-shaped flap.	Minimal web space deformity. Simple syndactyly often does not require grafting.	FTSG needed in walls of web space in complex syndactyly.
Braun et al. (2016) [48]	Flatt technique	Dorsal rectangular flap with a reverse T-shaped incision on the palmar side. Interdigitating zigzag flaps to separate digits, FTSG.	Not applicable	Web creep occurred in 26% of web spaces, with a significantly higher incidence in patients under two years old.
Braun et al. (2016) [48]	Dorsal omega flap	Dorsal omega flap with its apex extending to the level of the PIP joint. Anchor-shaped palmar incision.	Minimizes contracture formation.	Not applicable
Braun et al. (2016) [48]	Modified Flatt technique	Dorsal hourglass flap, interdigitating zigzag flaps with the most proximal being a hemi-triangular flap, FTSG.	Web creep reduced to 4.2% of web spaces compared with the original Flatt technique. No correlation between early surgery (<2 years old) and unfavorable outcomes.	Not applicable
Braun et al. (2016) [48]	V-Y and rectangular flap combination	Dorsal rectangular and volar V-Y triangular advancement flap to create web space with an S incision for digits. FTSG to cover raw spaces.	Simple and straightforward design. Minimizes scarring on the dorsal aspect of the hand. No contracture or web creep requiring revision.	Not applicable
Choong et al. (2024) [53]	Trilobed flap	A central lobe is used to reconstruct the web commissure, while two lateral lobes extend along the digits. The dorsal and palmar flaps interdigitate to minimize web creep. The flap is placed in the middle third of the MCP-PIP region.	Minimal web creep observed. Allows for better anatomical web-space reconstruction. Avoids palmar junctional scars, reducing web creep risk. Faster healing and reduced wound care burden.	Requires significant defatting for flap closure. Matching flap components is essential for proper fit. More interdigitating flaps may be needed in older children.
Barabás and Pickford (2014) [54]	Modified Flatt technique	The senior author employed the Flatt technique with a minor modification consisting of a dorsal hourglass-shaped web flap, interdigitating zigzag incisions, and full-thickness skin grafts for all but the mildest cases of syndactyly. The modification involved the transposition of narrow dorsal and volar triangular skin flaps carefully inset at the sides of the neo-commissure to minimize linear scarring. Skin grafts were initially taken from the groin but, after 2002, were sourced from the antecubital fossa. All operated hands were immobilized in long-arm splints with the elbow flexed at 90° for 18 days postoperatively.	The modification appeared to lower the incidence of web creep, possibly due to the zigzag flap configuration reducing the risk of contraction from linear scars. The method had a low complication rate, with graft failure occurring in only 4.9% of cases, and most resolving with conservative treatment. Infections were rare (2.8%) and successfully managed with oral antibiotics, without leading to graft loss. Hypertrophic scarring (3.5%) was self-limiting and did not contribute to web creep. The study found that most cases of web creep requiring re-release were associated with complex syndactyly or syndromic conditions.	Complications included a 4.2% rate of web creep, with 2.8% of cases requiring reoperation. One case of complete graft failure led to web creep in a child with Down syndrome due to repeated removal of dressings and hand-chewing behavior. The method required full-thickness skin grafting, which carries inherent donor-site morbidity. While the technique effectively reduced web creep, some cases in syndromic patients required multiple revision surgeries.
Karamese et al. (2016) [47]	Dorsal rectangular and volar V-Y flap	Dorsal rectangular flap and volar V-Y flap to create a natural web, minimizing web creep and contractures	Creates a natural web space with minimal web creeping and contractures. No ischemic injuries, flap loss, or neurovascular complications. High parent satisfaction scores. Simple and beginner-friendly design.	Hyperpigmentation occurred in two patients, but it was minor and did not impact function. One case of Grade 1 web creeping (Withey classification), which did not require revision surgery.
Dong et al. (2017) [49]	Dorsal double-wing flap	A double-wing flap was designed dorsally at the metacarpal heads, forming a triangle with a 60° angle at the intermetacarpal line. Zigzag incisions were made for digit separation, and a rhombic flap was created on the volar and dorsal sides. The flaps interdigitated to reconstruct the web space. The donor site was closed primarily without skin grafts.	No skin grafts required, minimizing complications like hyperpigmentation, scarring, and graft contraction. Web creep incidence was only 3%. No flexion contractures or necrosis observed. Good functional and cosmetic outcomes in 97% of patients. Simple and fast procedure.	One patient required revision surgery for web creep, but the correction was achieved without skin grafts. Study only encompassed short-term follow-up, so long-term outcomes remain uncertain.
Hsu et al., (2009) [41]	Modified V-Y dorsal metacarpal flap	Modified Sherif’s dorsal V-Y metacarpal flap by designing a full-thickness hexagonal island flap (3:1 length-to-width ratio) advanced volarly to form the new commissure. Triangular flaps in a zigzag pattern enabled complete digital separation. The flap, elevated with a subcutaneous pedicle, was sutured at its distal tip to the palmar skin. This technique was selectively applied to simple incomplete syndactyly cases with at least 2 mm of dorsal skin, assessed via caliper pinch test.	The modified V-Y flap enabled complete syndactyly release without the need for skin grafts in 53 percent of simple incomplete cases. There were no intraoperative complications, neurovascular compromise, or flap loss. The absence of a donor site for grafts minimized scarring and reduced operative time. The resulting commissure appeared natural, and most donor sites healed with minimal scarring.	This approach was not appropriate for complex or complete syndactyly, which typically required additional skin grafts to achieve adequate coverage. One early case experienced 5 mm of web creep, necessitating reoperation; later cases avoided this by advancing the flap 5 mm more proximally. The technique also carried a potential risk of vascular compromise due to pedicle torsion and could result in flap bulkiness at the pivot point.
Mericli et al. (2015) [46]	Tapered M-to-V flap	Under general anesthesia, an M-shaped dorsal and V-shaped volar flap were designed, with syndactyly released in a zigzag pattern. A cut-as-you-go technique ensured precise flap tailoring without vascular compromise. Flaps were inset with 5-0 chromic gut sutures, and hemostasis was achieved. Full-thickness groin grafts resurfaced sidewalls as needed. Postoperative care included dressings, immobilization, and staged follow-up.	The M-V flap approach allowed individualized flap planning tailored to each case of syndactyly, offering a high degree of anatomical accuracy. There were no flap losses and only partial graft loss in 10 cases, suggesting robust flap viability. The preservation of vascular integrity by avoiding undermining, combined with a well-structured postoperative protocol, supported wound healing. Complications were significantly lower in patients with shorter follow-up duration, and the majority of cases did not require surgical revision.	A total of 14% of web space reconstructions experienced complications, with web creep being the most frequent (9%), typically identified after a mean interval of 2.8 years. There were 10 partial graft losses, four cases of skin edge separation, three flexion contractures, and 10 surgical revisions. The complication rate increased with longer follow-up, reaching 28% in patients followed beyond two years, indicating a possible limitation in long-term durability.
De Smet et al., (1998) [55]	Flatt technique	The Flatt technique uses a large dorsal rectangular flap covering the web base and a reversed T-shaped palmar incision. Digits are separated with Z-incisions, and full-thickness skin grafts cover the lateral bare areas. Nailfolds are formed using the double pulpplasty method.	Web space pigmentation and scarring were normal or near normal in 64% of webs. Active abduction of at least 20° was obtained in 86% of webs. Normal flexion and extension in over 50 % of operative cases.	Web creep occurred in 26% of web spaces (n=13), with two needing surgical revision. Increased development of web creep in cases operated before the age of two years (11/23 vs. 2/27).
Guero (2019) [50]	Trident flap	Partial syndactyly release was performed using a trident flap without grafting. A V-shaped dorsal incision created three triangular flaps, while three 90° palmar cuts formed triangular and rectangular flaps. Flaps were elevated, rotated, and inset without undermining or nerve division, deepening the commissure with a natural, broad base. Minimal postoperative care was needed.	In most cases, the trident flap technique avoided full-thickness skin grafting, reducing donor site morbidity, scarring, and risk of graft failure. The absence of grafts also simplified postoperative care and minimized maceration risk. Surgical time was significantly shorter (mean: 16 min) compared to techniques requiring grafts (mean: 65 min). Functional and aesthetic outcomes were excellent, with satisfactory postoperative abduction (>30°) and minimal visible scarring, even in patients with darker skin. Web creep was rare (5%), and long-term follow-up (>15 years) showed stable results.	In most cases, the trident flap technique avoided full-thickness skin grafting, reducing donor site morbidity, scarring, and risk of graft failure. The absence of grafts also simplified postoperative care and minimized maceration risk. Surgical time was significantly shorter (mean: 16 min) compared to techniques requiring grafts (mean: 65 min). Functional and aesthetic outcomes were excellent, with satisfactory postoperative abduction (>30°) and minimal visible scarring, even in patients with darker skin. Web creep was rare (5%), and long-term follow-up (>15 years) showed stable results. The trident flap technique was limited to partial syndactyly cases that did not extend beyond the PIP joint. In cases where syndactyly extended distally, a full-thickness skin graft was required in at least one digit. Operating on two contiguous commissures in the same session was avoided to prevent excessive dorsal tension and impaired venous blood flow. Although web creep was minimal, two cases required revision surgery. Additionally, two hypertrophic scars (Grade 3) required corticosteroid treatment for satisfactory resolution.
Lumenta et al. (2010) [43]	Palmar and dorsal triangular flap	Used palmar and dorsal triangular skin flaps for commissure creation and multiple zigzag incisions for digit separation, with FTSGs from the groin.	Low rate of web creep, good scar quality, and favorable long-term functional outcomes.	High incidence of hair growth in grafted areas, occasional thickened scars, and pigmentation changes.
Yildirim et al. (2011)[44]	Dorsal separated V-Y advancement flap and volar triangular flap	The procedure involves making precise dorsal and volar incisions to create a V-Y advancement flap dorsally and a triangular flap volarly. The dorsal V-Y flap is carefully elevated while preserving its vascular supply, ensuring tension-free closure and minimizing scar contracture. The volar triangular flap is designed to provide additional coverage and optimize contouring of the interdigital space. Once the flaps are mobilized, the separated digits are meticulously closed, ensuring proper alignment and function. Full-thickness skin grafts may sometimes be applied to cover any bare areas. The surgical site is then dressed, and a postoperative splint is applied to maintain the corrected position during the healing phase.	No perioperative complications. No web creep. Excellent web function and aesthetic outcome. High patient satisfaction. Minimal scarring on the dorsum, which became inconspicuous within six months. Reliable vascular supply. Low risk of neurovascular injury. Applicable to both adults and children with simple, incomplete syndactyly	Requires careful dissection of neurovascular structures. In two cases, full-thickness grafts were needed for lateral bare areas. Postoperative splinting required for two weeks. Dorsal scarring may initially be visible before fading
Mallet et al. (2013) [56]	Dorsal T and omega flaps	The omega-flap involves a dorsal omega-shaped flap with a palmar anchor forming two lateral-palmar flaps. The dorsal flap is drawn with its base at the metacarpal heads and its apex at the PIP joint. Palmar incisions create an anchor extending from the PIP joint apex to just below the future web, with lateral branches ending at the mid-base of the adjacent digits. Once digits are separated using zigzag incisions, the dorsal flap tip is sutured to the palmar recipient site, and the lateral-palmar flaps are sutured to form the new web. The T-flap, described by Glicenstein, involves a dorsal T-shaped flap with its base at the metacarpal heads and its tip at the dorsal PIP. A palmar inverted T-incision is made, consisting of a longitudinal branch extending from mid-P1 to the future web level and a transverse branch at the web level. After separation with zigzag incisions, the dorsal flap tip is sutured to the palmar recipient site, with the web sides covered using full-thickness skin grafts.	High patient satisfaction (95%), low incidence of flexion contracture (3%), long-term preservation of web-space positioning in 83% of cases, subnormal DIP and PIP range of motion maintained, omega-flap associated with lower web retraction rates (4%) compared to T-flap (17%).	17% rate of web-creep at mid- and long-term follow-up, half required surgical revision with a second full-thickness skin graft, higher incidence of web retraction in the T-flap group, flexion contractures observed in 3% of cases, thickened junctional scars and dystrophic nails in some cases, hyperpigmentation at recipient sites in 22% of cases, and occasional hair growth at lateral aspects of separated digits.

Discussion

Embryology

Limb development begins in the fourth week of gestation from the limb field, with limb buds formed from mesenchymal cells surrounded by ectoderm [57,58]. The limb buds develop along three primary axes (anterior-posterior, dorsal-ventral, and proximal-distal), each regulated by distinct signaling centers and molecular pathways.

The anterior-posterior axis is controlled by the zone of polarizing activity (ZPA), which secretes sonic hedgehog (SHH) to induce polarization and establish a chemical gradient [59]. With the help of the apical ectodermal ridge (AER), the ZPA, located in the posterior mesoderm, maintains a constant source of SHH along the posterior aspect of the outgrowing limb [60].

The dorsal-ventral axis is established through a cascade involving WNT7a, Lmx1b, and Engrailed-1 (En1). WNT7a, located in the dorsal ectoderm, induces Lmx1b expression and thereby the dorsal aspect. While En1 expression, positively regulated by WNT and BMP signaling, restricts WNT7a expression to establish the ventral aspect [57].

The AER maintains the proximal-distal axis, which produces FGF8 and forms a positive feedback loop with mesodermal FGF10 to induce early limb growth [61]. Interaction of mesodermal FGF10 and ectodermal radical fringe induces ectodermal thickening of the limb bud's distal portion, delineating the ectoderm's dorsal and ventral aspects.

**Figure 5:**
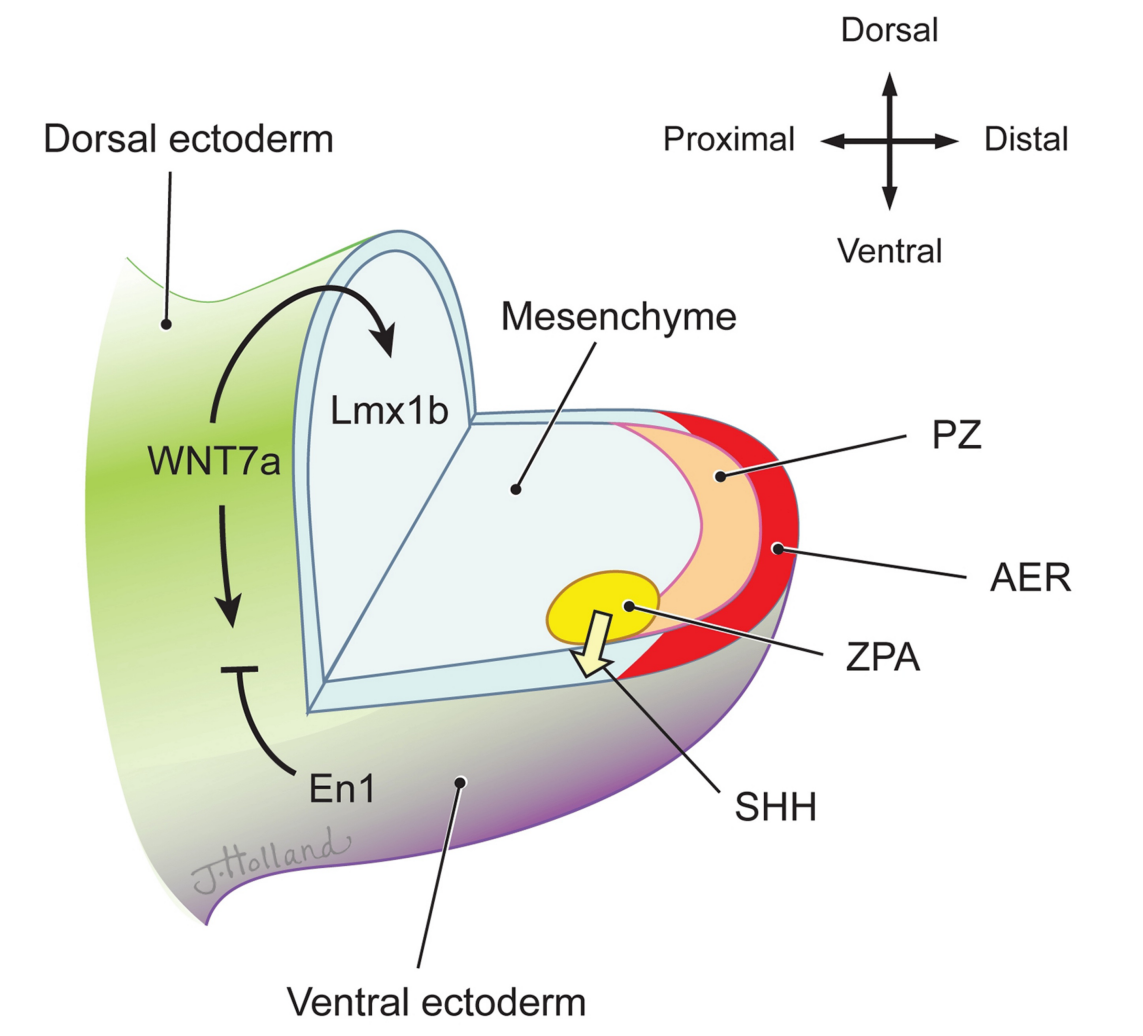
Gene regulation in limb development Image Credit: This original illustration was created by Jessica Holland MSMI CMI for the authors. This image was created using traditional drawing tools and then later rendered in Adobe Photoshop and labeled in Adobe Illustrator (© CC-BY-ND 2025 Jessica Holland MSMI CMI, St. George's University). ZPA: zone of polarizing activity, AER: apical ectodermal ridge, PZ: progress zone, SHH: sonic hedgehog. Arrows indicate progression/expression, black lines indicate inhibition

Around the fifth week of development, the hand plate forms where SHH and HOX gene family interactions determine digit number and identity [60]. SHH establishes an anterior-posterior BMP gradient, inducing interdigital webbing apoptosis and digit differentiation [60]. FGF suppression in the AER also contributes to apoptosis [60]. HOXD13 activates retinoic acid, leading to the formation of webbed spaces through apoptosis and extracellular matrix activation [52]. Conversely, mutations in HOXD13 can lead to disrupted retinoic acid signaling, affecting digit formation and associated syndactyly types, leading to persistent webbing characteristic of syndactyly [52]. It is worth noting that in a study by Ogino, teratogens, such as busulfan, can disrupt AER-related FGF expression, BMP4 expression, and ZPA-related SHH, leading to syndactyly-related malformations (Figure 5) [60,62].

Classification/Genetics

The classification of syndactyly has evolved from purely anatomical descriptions to more comprehensive clinical, genetic, and molecular approaches. One of the earliest records of syndactyly dates to the Middle Ages by Al-Zahrawi Abulcasis, an Andalusian surgeon. However, a formal classification system was not introduced until the turn of the 20th century. Roblot first proposed an anatomical classification in 1906, which was based solely on the physical presentation of syndactyly [14].

A significant advancement came in 1978 with the classification system developed by Temtamy and McKusick. Their model categorized syndactyly into five groups: simple, complex, acrosyndactyly, polydactyly-syndactyly, and symphalangism-syndactyly [63]. Over time, this classification model expanded to incorporate four additional syndactyly categories: interdigital, peripheral, central, and apical. Along with several subcategories, such as complete and incomplete subtypes and the bilateral versus unilateral subtypes [8,52]. These additions refined the classification system, allowing for more precise diagnosis and management planning [64]. However, even with these revisions, multiple syndromes with syndactyly being one of the clinical features are still exempted from this extensive classification system.

Many syndromic and non-syndromic types of syndactyly are associated with specific loci and proven to have mostly an autosomal dominant inheritance pattern. However, two of the later cases included in the model present as autosomal recessive, and one is transmitted as X-linked. Additionally, the types of syndactyly can be subdivided based on the specific characteristics of the fusion, including digits involved (fingers or toes), phalanges fused (distal, middle, or proximal), whether the fusion involves only soft tissues or also bones, and the extent of the fusion (complete or incomplete) [8,12-14].

Non-syndromic syndactyly: Non-syndromic syndactyly refers to congenital hand anomalies with digit fusion, classified into nine types. Type I, the most common, involves fusion between the third and fourth fingers or between the second and third toes. Type II syndactyly is heterogeneous and associated with polydactyly [14,65]. Types III-V exhibit specific digit and metacarpal fusion [14,16]. Types VI-VIII are rare, with unique features like unilateral syndactyly and uncertain inheritance patterns [8]. Type VII (Cenani-Lenz syndrome) involves complete synostosis of carpals, metacarpals, and phalanges, with possible forearm and lower limb abnormalities [14]. Type IX (mesoaxial synostosis syndactyly) shows complete syndactyly and severe bone reduction in the third and fourth digits [19]. This classification system aids in diagnosis and treatment.

Syndromic syndactyly: Syndromic syndactyly involves various genetic syndromes with distinct characteristics. Acrocephalosyndactyly syndromes (Apert, Apert-Crouzon, Carpenter, Jackson-Weiss, Pfeiffer, Saethre-Chotzon) feature cranial and limb malformations, with most involving FGFR mutations, except Carpenter syndrome (RAB23 gene) and Saethre-Chotzon syndrome (TWIST1 gene) [20]. Apert syndrome (MIM 101200) features craniosynostosis and hand syndactyly [21]. Apert-Crouzon syndrome includes maxillary hypoplasia and vertebral abnormalities [66]. Carpenter syndrome (MIM 20100) features acrocephaly, syndactyly, polydactyly, and brachydactyly [23]. Jackson-Weiss syndrome (MIM 123150) is characterized by syndactyly of the second and third toes. Pfeiffer syndrome (MIM 101600) has three subtypes, with Types II-III often fatal due to severe cranial defects [25]. Saethre-Chotzon syndrome (MIM 101400) presents syndactyly of the second and third fingers and third and fourth toes. Other syndromes include Goltz syndrome, EEC, and oculodentodigital dysplasia.

Clinical Diagnosis

Hand syndactyly can often be detected prenatally through ultrasound, especially when there are clear signs of digit fusion. However, detection can be challenging, particularly in cases where only soft tissue is involved. If prenatal detection occurs, a genetic review of potential carriers and identification of other associated anomalies can be conducted [8,67]. Additionally, conditions such as constriction bands, which may contribute to syndactyly, can sometimes be addressed with in-utero treatment. However, the risks associated with in-utero surgery for syndactyly often outweigh the benefits, especially given that syndactyly itself is typically not a life-threatening condition. Such interventions are generally reserved for severe cases, such as those involving multiple limbs or significant limb deformities [8,68]. In very complex cases, such as Apert syndrome, additional diagnostic measures like angiography may be employed to assess vasculature before surgery [69].

If syndactyly is not detected prenatally, it is usually evident at birth. Radiographs are commonly used to assess bone fusion, but a simple clinical maneuver can also help in identifying metacarpal or metatarsal fusion without the need for radiographic imaging. This maneuver involves attempting to separately displace two adjacent metacarpals in a ventral or palmar direction using the thumb and index finger [70].

Treatment Considerations

Surgical intervention for syndactyly, a practice established in the 19th century [9], is often recommended between 18-24 months for simple syndactyly and 12-24 months for complex cases to minimize complications from premature or delayed repair [2,71]. Surgery before six months is rare due to small digit size and anesthesia risks [72], and delays can impact hand function and psychosocial well-being [73]. The average age for repair is about 2.6 years [72,73], with potential long-term complications such as web creeping, which involves the appearance of abnormal webbing at the commissure between the digits [74].

Deciding on surgery involves assessing functional impairment, severity, and timing. Standard surgical steps include digit separation, addressing tissue and vascular structures, and resurfacing with flaps or skin grafts if needed [42,75]. Early intervention is crucial for syndactyly involving the thumb and index or small and ring fingers, due to the comparatively different lengths of the index and ring fingers, respectively. Early intervention prevents skeletal abnormalities [71,76] and more pronounced deformities in the thumb [71,77]. Surgery is ideally performed at 18 months (about one and a half years) for digits of equal length, with favorable outcomes in 75% of cases [10]. Since there is no difference in length, the formation of contractures and rotational deformities is reduced, allowing for a longer period where intervention may result in a favorable outcome. Multiple digit involvement may require staged surgeries to manage skin flap ischemia [5], as seen in Apert syndrome, where the third web space release is optional, and central digits of similar length may be deferred [78]. Unless there are marked arterial abnormalities or injury to the vessels, skin flaps are more at risk than the digits. Therefore, surgery is usually performed on one side of the middle digit at a time to reduce risks [5].

Grafts: Skin grafts are frequently used in syndactyly surgery due to the increased surface area of individual digits compared to conjoined ones, with individual digits having about 30% more skin [4]. Full-thickness grafts are preferred for their reduced tendency to scar and contract, although they may cause hyperpigmentation and hair growth, particularly when harvested from certain locations [10,79]. Split-thickness grafts, though less likely to cause hyperpigmentation, are associated with an eight-fold increased risk of web creep and skin instability [5]. Common donor sites include the lateral inguinal area, palmar wrist, antecubital fossa, and medial foot [10,42]. Different areas have been proposed to reduce the chance of hair growth after surgical intervention. However, complications with skin grafts remain a concern, including possible hair growth, donor site scarring, and potential darkening of grafted skin [76].

Recent studies have explored alternative grafting techniques to optimize functional and cosmetic outcomes. Mansoor et al. compared syndactyly release with and without skin grafts and found that the graftless group had significantly reduced operative time (47.1 ± 4.5 minutes vs. 90.5 ± 10 minutes), faster wound healing (15.2 ± 1.3 days vs. 22.5 ± 5.1 days), and earlier initiation of postoperative physiotherapy (20.6 ± 1.3 days vs. 27.7 ± 4.4 days). The findings support the increasing adoption of graftless techniques, which are associated with lower complication rates, earlier hand mobilization, and reduced postoperative care due to the absence of a donor site [80].

However, Woo et al. examined a novel approach using full-thickness skin grafts from the midline plantar foot, in contrast to the more commonly used groin or antecubital areas and graftless techniques. While “graftless” methods avoid donor site morbidity, they may leave noticeable dorsal hand scars. The study found that plantar full-thickness skin grafts resulted in better pigmentation matching postoperatively compared to groin grafts [81]. Woo et al. reported higher guardian satisfaction scores with both the hand (7.16 plantar vs. 5.05 groin, p < 0.001; 7.16 plantar vs. 4.36 no skin graft, p < 0.001) and the donor site (8.21 plantar vs. 6.30 groin, p < 0.001) when compared with the groin and graftless techniques for syndactyly release. However, considerations for plantar grafts include a higher risk of infection, whereas groin grafts are more prone to wound dehiscence postoperatively. The gradation skin graft from the plantar instep has thus emerged as a promising option, balancing aesthetic advantages with functional outcomes and reduced risk of complications [82].

Flaps: Flaps are critical for reconstructing the web space in syndactyly repair. Preferred techniques include the dorsal rectangular flap, dual triangular flap, and dorsal pentagonal local flap [42,45]. For simple incomplete syndactyly, a three-flap webplasty suffices without the need for skin grafting [10,83]. Other graft-free methods include the trilobed flap, three-square-flap, and modified bell-bottom flap [48]. When the thumb and index fingers are involved, a four-flap Z-plasty or a central V-Y with lateral Z-plasty is commonly used [6,10,84,85]. Techniques for separating the lateral digits include the zigzag technique, which is preferred for its ability to reduce bone exposure and avoid longitudinal scarring [9,10].

Mallet et al. described two dorsal flap techniques, the T-flap and the omega flap, both used in conjunction with full-thickness skin grafts. The study found no significant difference between these cohorts in operative time, healing, or the development of contractures and digit deformities. However, the T-flap group had a significantly higher incidence of web creep (17% vs. 5%) than the omega flap group [56]. Additionally, complex syndactyly release cases were more likely to develop clinodactyly postoperatively compared to simple syndactyly cases, suggesting that the severity of syndactyly plays a role in postoperative digital alignment outcomes.

An additional technique described by Lohmeyer et al. involves the use of a soft tissue distractor that is inserted into the bone and separates syndactyly in patients where manual surgical separation is not feasible. Unlike the graftless Pennig fixator technique described by Li et al., Lohmeyer et al. utilized this method in 168 patients before undergoing syndactyly repair with grafts. The study reported no cases of ischemia or surgical revision and only five cases of infection during hospitalization [86,87]. This technique has demonstrated significantly improved functional and aesthetic outcomes, with the added benefit of potentially reducing the need for grafts in syndactyly repair.

Barriers to Reconstruction in Complex Cases

Surgical reconstruction in complex syndactyly cases presents unique challenges due to vascular considerations, risk of web creep, and higher revision rates [76]. Goldfarb et al. reported that 48% of patients developed postoperative web creep, while 24% required revision surgeries, approximately twice the reoperation rate observed in patients with simple, skin syndactyly [88]. Most reoperations were performed within 14 months postoperatively, typically for contracture release, highlighting the increased likelihood of complications in complex syndactyly cases.

Proper separation of conjoined digits is crucial, particularly when digital nerves and arteries bifurcate near the new commissure [10]. In cases of three-digit fusion, border digit arteries may be ligated due to their secondary supply [10]. Some surgeons recommend a staged approach to mitigate complications such as ischemia and flap necrosis, separating digits sequentially to preserve perfusion. A novel quadrangular flap technique has been employed in Apert syndrome Type III, utilizing first commissure and fourth webspace flaps to reduce tension, with skin grafts covering raw surfaces [51].

For nail fusion, the nail bed, matrix, and nail are divided, with slight excess removal to facilitate reconstruction [10]. For nail fusion, techniques such as triangular opposed flaps, rectangular local flaps, or palm-raised flaps facilitate nail bed reconstruction [42].

Graftless Techniques

Recent advancements in surgical techniques have introduced several "graftless" methods for syndactyly release, minimizing the risks associated with skin grafting. These techniques are particularly useful for simple syndactyly and cases with predictable anatomy. "Graftless" techniques are associated with decreased occurrence of hyperpigmentation, web creep formation, and hypertrophic scarring [54].

The trilobed flap technique utilizes skin from the dorsum of the hand and fingers to close raw borders, eliminating the need for a distant donor site. This technique involves careful planning of skin incisions to maximize available skin and allow for neurovascular structure visualization. The trilobed flap is placed between the MCP and PIP joints in the middle third, with dorsal interdigitating zigzag flaps extending to the PIP and DIP joints [53].

In complex cases, such as ulnar longitudinal deficiency, a combined dorsal and palmar V-flap technique with skin grafting may be necessary [53]. Alternatively, the dorsal hexagon local flap technique involves designing a hexagon flap on the dorsal proximal interdigital region and advancing it distally and volarly to reconstruct the web space without grafting [24].

The Pennig fixator is another graftless approach that expands the web space without skin grafting. This method uses an external separation device for tissue expansion and subsequent Z-plasty in a second procedure, minimizing complications like skin necrosis and reducing the need for skin grafts. This technique leverages the strain-stress principle to develop sufficient tissue for reconstruction. It is indicated for both simple and complex syndactyly, though it is less suitable for children under two years old [87].

Additionally, Jung et al. and Fangxing et al. described the use of synthetic dermal substitutes, Integra and Pelnac, respectively, as graftless methods in syndactyly repair [89,90]. These substitutes are particularly beneficial in complex cases or repairs extending to the distal digit, where local flap mobilization may be insufficient. These techniques avoid donor site morbidity and the need for postoperative hand immobilization, as seen in groin grafts. They also reduce operation time and minimize complications such as wound dehiscence, flap ischemia, or necrosis. Patients treated with these methods reported improved range of motion and aesthetic outcomes, with no hypertrophic scarring and near-normal pigmentation postoperatively.

Landi et al. further contributed to graftless techniques by introducing a hyaluronic acid scaffold in syndactyly repair. At a two-year follow-up, 22 of 23 patients exhibited no contractures, web creep, or secondary deformities, with only one case of Grade 4 web creep. Patients displayed minimal scarring, with near-normal pigmentation and good postoperative functional mobility. No hypertrophic scarring or keloids were observed. However, at the three-week postoperative follow-up when the scaffold was removed, three patients exhibited areas of hypergranulation due to scaffold shifting [91]. These cases were managed conservatively with topical steroid cream. Healing with dermal substitutes was typically completed within 37 days postoperatively. Synthetic dermal substitutes provide a durable and long-lasting solution, promoting optimal wound healing and favorable outcomes regarding web creep and scar appearance, minimizing the need for additional surgeries [92].

Outcomes

Outcomes following syndactyly repair vary depending on the type and complexity of the malformation. Complex syndactyly often results in poorer motion and higher rates of reoperation.

Surgical outcomes: Patient-reported outcomes generally show higher satisfaction with upper extremity function, peer relations, and reduced pain interference than parent proxy reports. Post-reconstruction functionality is usually comparable to that of healthy children across physical health, emotional functioning, school performance, and psychosocial health. However, minor impairments in upper extremity function may persist, especially in patients with developmental delays or comorbidities [93]. According to Goodenough et al., the overall 30-day readmission rate is low at 0.68%, with higher rates observed in complex syndactyly repairs (2.1%) compared to simple repairs (0.8%) [94]. Surgical site complications are the most common reason for readmissions, but pediatric hand surgery generally has a low risk of 30-day readmission.

Technique-specific outcomes: Barabás and Pickford operated on 144 patients with Poland syndrome using a modified Flatt’s technique also known as an hourglass dorsal advancement flap, which is a technique using a dorsal, hourglass flap coupled with an interdigital zigzag technique with or without a full-thickness graft obtained from the groin or antecubital fossa. Only 17 patients had adverse outcomes in the five-year follow-up; 4.9% of patients had graft failure, all managed conservatively (two had full graft failure), and 2.8% required reoperation [54]. Compared to the Flatt technique and other techniques that utilized the T-incision, the modified Flatt technique had better postoperative outcomes, with only 4.2% developing web creep compared to the 26% reported by De Smet et al. using the Flatt technique [55].

For Apert syndrome Type III, postoperative complications may include deviation of the index finger and clinodactyly of the thumb, though these are less severe compared to Type I hands. The most serious complication in these cases is digit infection, which may result in edema and vascular compromise [51]. The trilobed flap technique has shown promising outcomes with faster healing times and reduced dressing needs, with no increased adverse effects compared to traditional graft techniques [53]. Similarly, the gradation skin graft technique offers excellent cosmetic results with improved color and texture match, provided the graft is carefully designed and placed [82].

The dorsal hexagon local flap technique has also been effective, yielding satisfactory results with no complications such as flap necrosis or the need for additional skin grafting. It restores normal finger contour without lateral flexion or web creep [24]. However, skin grafting may result in higher rates of postoperative complications compared to techniques using local flaps without skin grafts.

The Pennig fixator technique demonstrated excellent outcomes with no infections, skin necrosis, or nonunion and satisfactory final appearance and digit function. It avoids skin grafting and reduces the risk of blood vessel damage, offering an efficient and effective approach for syndactyly repair [87]. Synthetic dermal substitutes provide efficient coverage of skin deficits with favorable outcomes in web creep and scar appearance. However, the rate of integration and healing rates are still under evaluation; they do not fully eliminate the need for skin grafts [92].

Outcome Evaluation

The Withey Score is a reliable and validated tool for evaluating syndactyly repair outcomes. It provides a comprehensive assessment of both hand function and cosmesis, demonstrating good interobserver and intraobserver reliability while correlating well with patient satisfaction [95]. Compared to the Vancouver Scar Scale (VSS), the Withey Score is more specific for syndactyly repair outcomes, except for scar appearance, where the VSS provides additional detail [95]. Furthermore, both scoring systems allow for independent evaluation by surgeons or other qualified professionals, ensuring objective postoperative assessments. The mean scores from these systems can also be used for group comparisons or longitudinal tracking of patient outcomes, making them valuable tools for both clinical practice and research.

Several studies included in this review utilized the Withey Score and VSS to evaluate postoperative outcomes in patients undergoing syndactyly repair. Specifically, Choong et al., Kurebayashi et al., Barabás and Pickford, and Mallet et al. employed the Withey Score to assess the development of web creep postoperatively in their cohorts. This scoring system evaluates five postoperative parameters, including scar quality, presence of flexion-extension deformity, web creep, lateral flexion deformity, and rotation deformity, using a four-point scale (0-4), where a score of 0 indicates normal web healing and a score of 4 represents significant web creep. Additionally, Li et al. used the VSS to evaluate postoperative scarring in newly formed web spaces. The VSS assesses pigmentation, erythema, thickness, and scar pliability using a five-point scale (0-5), where 0 indicates normal healing and 5 denotes severe scarring. Notably, studies such as Fangxing et al., Guero, Karamese et al., Landi et al., Goldfarb et al., and Lumenta et al. incorporated both scoring systems to provide a more comprehensive evaluation of postoperative outcomes, including range of motion and patient-reported satisfaction.

## Conclusions

Hand syndactyly research has made significant progress, advancing from early anatomical studies to a deeper understanding of its genetic and embryological causes. The classification system has expanded from five to nine primary types, but many cases remain unclassified, indicating a complex etiology. While some embryological defects are well understood, complete elucidation of all causative factors remains ongoing. Genetic research has provided valuable insights, but many cases lack definitive explanations. Surgical techniques have improved over time, with newer methods aiming to reduce complications associated with traditional approaches. Ongoing research and long-term studies are crucial to refine diagnosis, improve treatment outcomes, enhance diagnostic accuracy, and achieve better overall patient care. Further investigation is necessary to fully understand hand syndactyly's etiology and develop effective management strategies.
